# Immobilization of Lipase on Silver Nanoparticles via Adhesive Polydopamine for Biodiesel Production

**DOI:** 10.1155/2014/389739

**Published:** 2014-09-10

**Authors:** Kanchana Dumri, Dau Hung Anh

**Affiliations:** ^1^Department of Chemistry and Materials Science Research Center, Faculty of Science, Chiang Mai University, Chiang Mai 50200, Thailand; ^2^Research and Development Institute/Department of Chemistry, Faculty of Science and Agricultural Technology, Rajamangala University of Technology Lanna, Chiang Mai 50300, Thailand

## Abstract

Biodiesel production technology is competitive in terms of low cost and alternative source of energy which should be not only sustainable but also environmentally friendly. Designing of the lipase immobilization for biodiesel production has a remarkable impact and is still challenging. In this work, biodiesel production from soybean oil was enhanced and facilitated by using a novel biocatalyst consisting of commercial lipase (EC 3.1.1.3), silver nanoparticles, and polydopamine. Silver nanoparticles (AgNPs) were synthesized with a size range of 10–20 nm. Polydopamine (PD) was delivered by the self-polymerization of dopamine in 10 mM Tris-HCl pH 8.5 and simultaneously coated the AgNPs to form a PD/AgNPs complex. Lipase was immobilized on the PD/AgNPs complex surface via covalent bonds to form a tailor-made biocatalyst consisting of immobilized lipase/PD/AgNPs complex (LPA). The formation and morphology of each composition were characterized by UV-Vis spectroscopy and scanning electron microscope (SEM). Significantly, gas chromatography analysis showed a remarkable biodiesel production yield of 95% by using the LPA complex at 40^°^C for 6-hours reaction time, whereas the yield was 86% when using free lyophilized lipase. The LPA complex was apparently reusable after 7 batches and the latter conversion rate of soybean oil was decreased by only 27%.

## 1. Introduction

Biodiesel production from vegetable oil has been increasing in the last decade in ASEAN countries, for example, Thailand, Malaysia, Indonesia, Vietnam, and The Philippines, since many of them have diverse and high yield crops of oleaginous plants, for example, palm, soybean, and sunflower [1, 2]. This is relevant to the world's rapid industrialization and increasing population [3]. For example, since 2010, Thailand had already thirteen active biodiesel producers with a capacity of 1970-million-liter biodiesel B100 or unblended biodiesel. In the year 2012, biodiesel consumption increased by 35% [4]. Transesterification of vegetable oils by short chain alcohols (methanol or ethanol) catalyzed by lipase (EC 3.1.1.3) is a worldwide biochemical mechanism to produce biodiesel [5] and there are two modes of lipase usage: free or soluble lipase and immobilized lipase. In early 2001, using free lipases from* Candida rugosa* and* Pseudomonas cepacia* along with methanol as acyl acceptor, a method was reported to yield biodiesel from soybean oil up to 80 and 90%, respectively [6]. However, free lipase based technology is costly and the reaction rate is low [7, 8]. Immobilized lipase methods have been introduced to improve lipase stability and reusability. Herein immobilized enzymes are defined as “enzyme physically confined or localized in a certain defined region of space with retention of their catalytic activities, which can be used repeatedly and continuously” [9]. Employed lipases for immobilization have been derived from microorganisms to animals, for example, from pancreatic porcine,* Burkholderia cepacia*,* Pseudomonas* spp., and* Candida* spp. [8, 10]. Immobilization methods are also various, for example, adsorption on acrylic resin, celite, and anion resin, covalent bonding using silica-PVA and styrene-divinylbenzene, entrapment using hydrophobic sol-gel support, and cross-linking using glutaraldehyde [10]. These methods can yield biodiesel from soybean oil using adsorbed lipase [11] and from mahua oil using cross-linked lipase [12]. However, these techniques are still sophisticated and require multiple-steps material consumption in case of* Pseudomonas cepacia* cross-linked lipase [12], as well as time consumption for oil transesterification (49 hour) in case of absorbed* Pseudomonas fluorescens* lipase on macroporous polypropylene matrix [11].

In recent years, dopamine, an adhesive protein from mussel, has been found and applied for multifunctional coatings in surface [13, 14]. Dopamine contains catechol and amine functional groups. It can self-polymerize in alkaline environments to form thin adherent polydopamine (PD) on a wide range of materials including noble metals (e.g., Ag and Au) and iron oxide [13–15]. Polydopamine has also been studied for enzyme immobilization, for example, trypsin [16], glucose oxidase [17], and lipase [18]. Enzymes can be covalently immobilized on a polydopamine surface via nucleophiles [19]. On the other hand, silver nanoparticles (AgNPs) have been known to exhibit nonspecific antibacterial property in silver-based biomolecule coatings [20–23]. Since biodiesel production technology is competitive in terms of low cost and reusability of materials, ASEAN countries are increasingly producing biofuels. To this end, we carried out the synthesis of a novel biocatalyst consisting of porcine pancreatic lipase/polydopamine/silver nanoparticles. Here we describe its apparent biochemical and morphological properties and its application in biodiesel production from the soybean oil which is the one of the dominant productivity of oil plant in the Northern Thailand [24, 25].

## 2. Materials and Methods

### 2.1. Materials

Porcine pancreatic lipase (EC 3.1.1.3), dopamine hydrochloride, standard fatty acids, and fatty acid methyl ester were purchased from Sigma-Aldrich (Germany). Other chemicals were of analytical grade. Soybean oil was obtained from commercial supplier (Chiang Mai, Thailand).

### 2.2. Synthesis of Biocomplex Consisting of Immobilized Lipase, Polydopamine, and Silver Nanoparticles (LPA Complex)

AgNPs of size 2–20 nm were synthesized as described by Solomon et al. (2007) [26]. 20 mL of colloidal AgNPs solution was mixed well with 20 mL of Tris-HCl buffer 20 mM (pH 8.5). Afterwards, 100 mg dopamine hydrochloride was added stepwise (approximately 2 mg/15 s) to a vigorously stirred AgNPs solution at room temperature for 1 hour to form polydopamine/AgNPs complex (PD/AgNPs). Afterwards, 40 mL of PD/AgNPs mixture solution was added dropwise (100 *μ*L/15 s) to a vigorously stirring 40 mL lipase solution (100 mg lyophilized lipase and sodium phosphate buffer 10 mM, pH 7.0) for 3 hours, 180 rpm at 4°C. The formation of an amorphous immobilized lipase/PD/AgNPs complex was observed immediately after addition of lipase solution. The LPA complex was collected for centrifugation and stored at 4°C prior to further experiment. A polydopamine/AgNPs complex without enzyme was used as negative control. For the immobilization yield, supernatant was taken for lipase activity measurement as described below and protein concentration was determined as described by Bradford [27].

### 2.3. Enzyme Assays for Soluble Lipase and Immobilized Lipase

Lipase activity was determined spectrophotometrically at 232 nm following the hydrolysis of 1% (w/v) Tween 80 as substrate in Tris-HCl buffer (20 mM, pH 8.5) containing 80 mM CaCl_2_ (designated as substrate solution). Reaction was carried out at 25°C (modified from [28]). The activity of authentic soluble lipase (1.25 mg/mL in 10 mM sodium phosphate butter pH 7.0) was measured by adding of 100 *μ*L of enzyme solution to 900 *μ*L reaction mixture consisting of 800 *μ*L sodium phosphate buffer and 100 *μ*L substrate solution. The hydrolysis rate of Tween 80 was directly monitored by measuring the decrease of absorbance at 232 nm over 10 min [28].

For the determination of immobilized enzyme activity, 1 mL LPA complex in a 2 mL Eppendorf tube was centrifuged at 5000 rpm for 5 min. Supernatant was replaced by 1 mL of deionized water and the pellet was vortexed for 1 min at 180 rpm for washing. This washing step was repeated 3 times and then the pellet was mixed with 1 mL reaction mixture containing 100 *μ*L substrate solution and 900 *μ*L sodium phosphate buffer (10 mM, pH 7.0). The reaction mixture was vigorously vortexed at 180 rpm for 10 min at room temperature. Afterwards, it was centrifuged at 5000 rpm for 5 min and 100 *μ*L of supernatant was dissolved in 900 *μ*L sodium phosphate buffer. The absorbance of the latter was measured at 232 nm and related to the activity of immobilized lipase in the LPA complex by comparison with authentic soluble lipase activity.

For assessment of enzyme immobilization yield, 1 mL LPA complex in a 2 mL Eppendorf tube was centrifuged at 5000 rpm for 5 min. Afterwards, 100 *μ*L of supernatant was taken for soluble lipase activity measurement as described above.

### 2.4. UV-Vis Spectroscopy Study

UV-Vis spectra of colloidal silver nanoparticles, PD/AgNPs, and immobilized lipase/PD/AgNPs complex were recorded in the range of 200–800 nm using a Lambda Bio 20 UV Spectrophotometer (Perkin Elmer, USA). For these experiments, 50 *μ*L of sample was mixed well with 950 *μ*L sodium phosphate buffer (10 mM, pH 7.0).

### 2.5. Characterization of AgNPs, PD/AgNPs, and LPA Complexes by Scanning and Transmission Electron Microscopy (SEM and TEM)

The morphologies and dimensions of samples were characterized by SEM and TEM (JEM 5910 LV and JEM 1010 JOEL Ltd., Japan) with accelerating voltage of 15 kV and 80 kV, respectively, with fit magnification.

### 2.6. Study on Optimal Temperature and Thermal Stability of Soluble and Immobilized Lipase

The temperature dependence of immobilized lipase activity was investigated in temperature range from 30 to 55°C in comparison to soluble lipase and activities of both forms were determined as described in Section 2.3. The thermal stability of immobilized lipases was investigated by determining its activity at optimal operating temperature after incubation period at 0, 2, 4, and 6 hours [29].

### 2.7. Biodiesel Production

Reaction was performed in a 100 mL Duran bottle containing 5 g soybean oil and 15 g MeOH. For the conversion of soybean oil, MeOH was added at the reaction times 0, 2, and 4 hours (5 g each time, i.e., 15 g in total). This stepwise addition of MeOH (3 × 5 g) to reach an oil to MeOH ratio of 1 : 3 (w/w) was implemented because this strategy has been widely proposed for biodiesel production studies [10]. An appropriate amount of immobilized lipase/PD/AgNPs (i.e., pellet LPA complex that was collected after centrifugation) or lyophilized lipase was applied. The reaction mixture was continuously stirred at 120 rpm for 6 hours at 40°C (optimal conditions). For reusability tests, the reaction mixture was centrifuged at 5000 rpm for 30 min at 4°C to collect the immobilized lipase pellet and the pellet was then reused with fresh substrates for further batch of biodiesel production.

### 2.8. Gas Chromatography Analysis of Soybean Oil

Soybean oil was identified using a HP6890 gas chromatograph equipped with a capillary column (AT-5, 30 m × 0.25 mm I.D. × 0.25 *μ*m film thickness) and fitted with FID detector. The column temperature was increased from 150°C to 300°C at a rate of 4°C/min and maintained for 3 min at 300°C. Fatty acids were calculated by comparing the retention times and peaks between sample and authentic standards (palmitic acid, stearic acid, oleic acid, linoleic acid, and linolenic acid; Sigma-Aldrich).

### 2.9. Fatty Acid Methyl Esters (FAMEs) Analysis (Biodiesel Products)

0.5 mL of reaction mixture was mixed with 1.0 mL isooctane for 2 min. Following centrifugal separation, the organic upper layer was collected and washed twice with distilled water and dried over anhydrous Na_2_SO_4_. The solvent was dried under N_2_ steam and dissolved in 0.25 mL of CH_2_Cl_2_. The previous GC condition of soybean oil analysis was applied. FAMEs formations were analyzed by means of authentic fatty acid methyl ester standards (methyl palmitate, methyl stearate, methyl oleate, methyl linoleate, and methyl linolenate; Sigma-Aldrich).

## 3. Results and Discussion

### 3.1. Characterization of AgNPs, PD/AgNPs, and LPA Complexes

**Figure 1:**
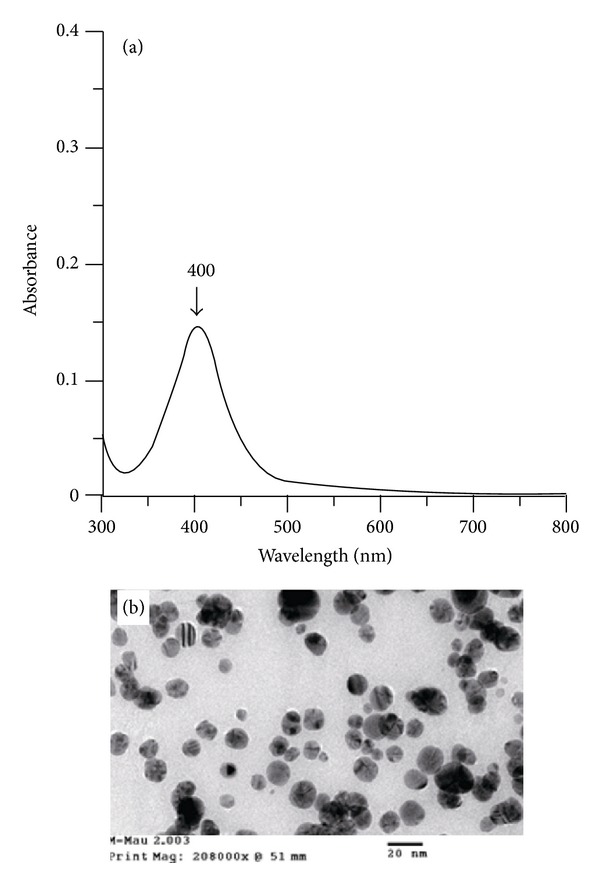
Typical UV-Vis spectrum (a) and TEM image (b) of silver nanoparticles.

The AgNPs, which were synthesized by NaBH_4_, were selected by the following reasons: (1) they can be used as a supporting core for the lipase immobilization to enhance the stability of lipase and to build morphologically controllable particles; (2) their size could be controlled, from 10 to 20 nm in diameters, so the smaller size particles will enlarge the total acting surface of reacting silver particles in reaction mixture. Obviously, this would lead to higher binding amount and volume of polydopamine and therefore the silver nanoparticle/polydopamine complex can trap lipase molecules massively; (3) colloidal AgNPs are not precipitated and agglomerated; hence they facilitate the uniform coating of polydopamine, and (4) they contribute to the probable antibacterial property of the biocatalyst as described in works where AgNPs were used to produce materials with antibacterial properties, for example, nanocomposites [30, 31]. Herein, AgNPs synthesis was modified from previous report [26] by increasing the [NaBH_4_] to [AgNO_3_] ratio to 10 : 1 to oxidize the AgNO_3_ completely to Ag. The colloidal AgNPs were yellowish and had a characteristic absorption spectrum with one maximum at 400 nm (Figure 1(a)). The TEM image in Figure 1(b) reveals the high density of the colloidal AgNPs sample. Particle sizes are approximately 10–20 nm.

For the next step to form the PD/AgNPs complex, the self-polymerization of dopamine in alkaline pHs [14] can form a thin polydopamine layer on noble metals [15]. Polydopamine has a robust polymerized aromatic structure and its formation is influenced by operating temperature, pH, and concentration of the dopamine solution [32]. In our experimental design, dopamine was selectively used to coat AgNPs to generate the PD/AgNPs complex, which were greenish black. In addition, previous works on coating dopamine have also suggested that an antibacterial surface was created by AgNPs composite [20, 23, 33]. Therefore, we assume that the presence of surfaced AgNPs herein alternatively protects the active lipase from bacterial degradation in different performance [34, 35] and probably allows the immobilized lipases to remain their activities for further approach.

**Figure 2:**
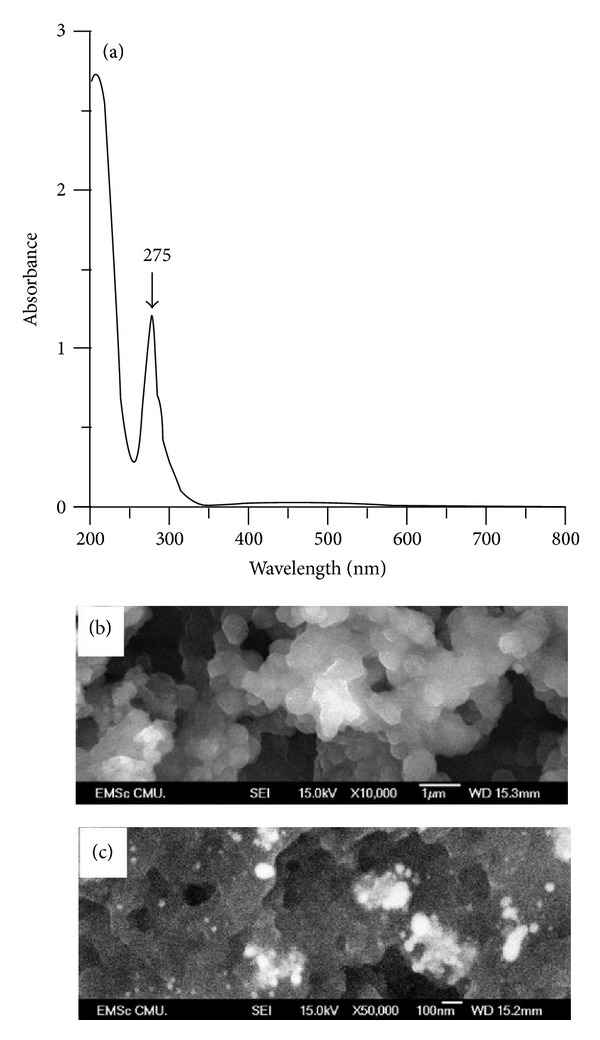
Typical UV-Vis spectrum (a) and SEM images ((b), (c)) of polydopamine (PD) and silver nanoparticles (AgNPs) complex.

The absorption spectrum behavior of PD/AgNPs (Figure 2(a)) has one distinguishable maximum at 275 nm and no representative peak for AgNPs at 400 nm. It can be deduced that AgNPs were covered and entrapped by PD and thus could not be detected by UV-Vis spectroscopy. SEM image in Figure 2(b) shows the PD/AgNPs agglomerates with size of approximately 200 nm. Diverse surfaces sizes of polydopamine were reported with sizes from 4 nm to over 100 nm by means of thin film [20, 32, 36].

Figure 2(c) reveals the bright spots clustered on a dark grey matrix, which are represented for AgNPs. These demonstrate that AgNPs could be partly captured on the surface of the PD/AgNPs complex. By means of capturing AgNPs on the PD/AgNPs complex surface, the PD/AgNPs complex could possibly exhibit antibacterial growth property [20, 31].

The most important step was for immobilized lipase to form the complete complex with PD/AgNPs. Lipase from porcine pancreas was immobilized on the PD/AgNPs complex via covalent bonds between the active quinone groups of PD and the amine and thiol groups of lipase [14, 19]. The polymerization of dopamine generates active residual quinones which allow covalent immobilization of the protein via Michael addition of cysteine (Cys), histidine (His), and lysine (Lys) residues of the lipase from porcine pancreas as well as Shiff base formation [14, 19, 36]. Thus, there is no need to use coupling reagents (e.g., glutaraldehyde or (3-aminopropyl) triethoxysilane) which require sophisticated procedures and may influence the protein structure [10, 37, 38].

**Figure 3:**
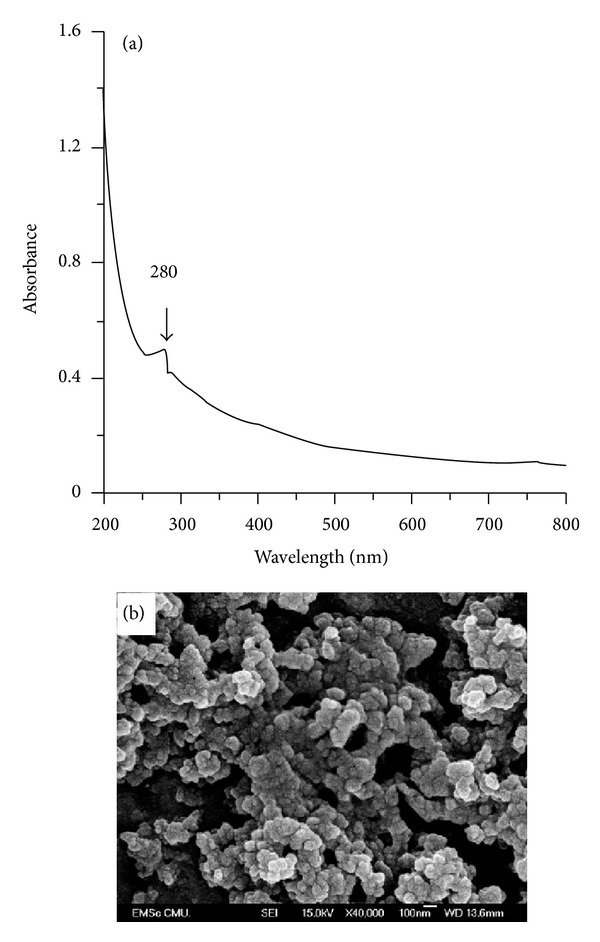
Typical UV-Vis spectrum (a) and SEM images (b) of immobilized lipase adhesive with PD/AgNPs complex.

In this work, a black precipitate was formed immediately after addition of lipase to the PD/AgNPs solution. The absorbance spectrum of the lipase-immobilized PD/AgNPs complex solution has a unique maximum at 280 nm (Figure 3(a)). The lipase immobilization on the PD/AgNPs complex with a ratio of lipase/PD of 1 : 1 (w/w) changed the typical spectrum of the PD/AgNPs complex. The high background in the spectrum possibly resulted from the black particles in the sample solution. The loaded lipase is assumed to be immobilized entirely onto the surface of the PD/AgNPs complex. The SEM image in Figure 3(b) demonstrates the homologous morphology of the lipase/PD/AgNPs agglomerates with an average size of 50–100 nm and they are not similar to the PD/AgNPs complex. The formation of separate particle chains can imply that the immobilization of lipase sharpens the PD/AgNPs complex from 200 to smaller 100 nm blocks by uncertain mechanisms. The spherical morphology of the LPA complex may be caused by the SEM sample preparation. On the other hand, this obtained LPA shape might contribute to the hydrolysis yield of vegetable oil by means of its random porous and interfacial structures. It might help to shear oil droplets to smaller sizes during transesterification that takes place under vigorous stirring and heating condition. It might also increase the access and exposure of lipid substrate to active site of immobilized lipases and lipid substrate molecules would be retained in porous matrix to react with the interfacial lipase in an appropriate time.

### 3.2. Fast Lipase Immobilization and Hydrolyzing Activity of Immobilized Lipase

The apparent protocol for immobilization allows the complete immobilization when the loading lipase/dopamine weight ratio was 1 : 1 and concentrations of both loading lipase and dopamine were 1.25 × 10^−3^ (w/v) in a total reaction volume of 80 mL. In the aqueous phase that was collected after centrifugation of the LPA complex solution, we could observe no lipase activity toward hydrolyzing of Tween 80 or it might be too low to detect enzyme activity. On the other hand, Bradford test of the residue of loading lipase protein after immobilization showed that 92 mg loading lipase took part in immobilization when 100 mg dopamine was used to cover a given AgNPs. Taken into consideration, this technique gives higher immobilization yield in comparison to 60–85% in case of covalent bond based immobilization using polyglutaraldehyde activated styrene-divinylbenzene (STY-DVB-PGA) for lipase from* Thermomyces lanuginosus* [39, 40] and silica-polyvinyl alcohol for lipase from* Burkholderia cepacia* [41]. As other heterogeneous biocatalysts, not only the mass transfer limitation but also the ratio of enzyme/support matrix (w/w) is taken into consideration. In our experiment the ratio of lipase/dopamine (i.e., lipase/support matrix; w/w) was to 1 : 1, whereas it was approximately 1 : 5 when using *β*-cyclodextrin/hexamethylene diisocyanate [42] and 1 : 100 when using STY-DVB-PGA [41]. This may have physically increased the exposure of immobilized lipase to substrates and enhanced the catalytic property of the LPA complex. In addition, the instant self-polymerization of dopamine curtails the time consumption. The entire immobilization process herein required 4 hours. In comparison, previous reports required up to 24–30 hours [37, 41, 43]. Hence, this can reduce the degeneration of the lipase while immobilizing occurs. The correlative lipase activity of the LPA complex (i.e., immobilized lipase) was assumed as approximately 25.2 U/mg LPA complex in comparison with 23.9 U/mg lyophilized lipase (dimension U/mg materials). In other words, a given polydopamine-immobilizing lipase quantity might have approximately 2.2-fold higher activity in comparison to similar quantity of lyophilized lipase towards Tween 80 as substrate in this work. Related to the described morphology of LPA complex (Figure 3(b)), previous work reported the interfacial* Candida rugosa* lipase giving a higher activity than in the bulk [44]. Thus, a possibly similar mechanism might have occurred in our case study.

The use of polydopamine gives not only the higher immobilization efficiency but also the activity of bound enzyme, and it was in concurrence with a report elsewhere [18]. We achieved an immobilized lipase on the PD/AgNPs complex and this biocatalyst complex remarkably demonstrated higher hydrolyzing activity than previous reports [42, 45]. This can be related to different immobilization techniques, for example, adsorption, entrapment, and polymer-based supports, for example, natural polymers such as alginate, chitosan, or polydopamine, synthetic polymers such as polyvinyl chloride and polyaniline [10], and inorganic materials such as silica, glass, or activated carbon [18, 42, 45].

### 3.3. Optimal Temperature and Thermal Stability

**Figure 4:**
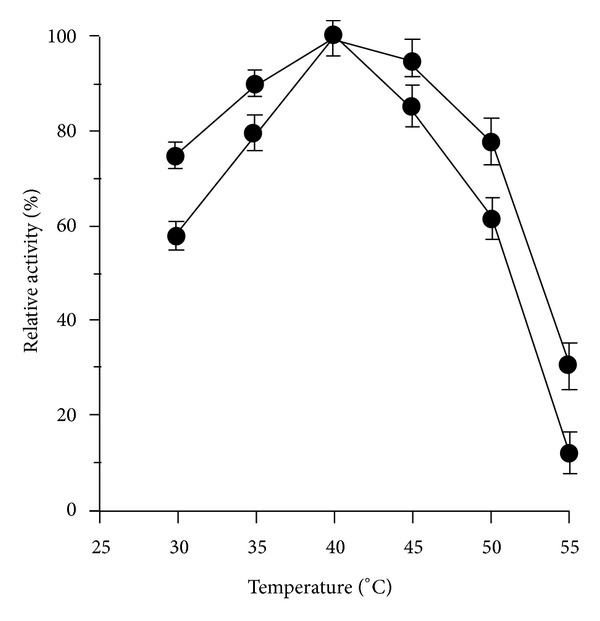
Influence of temperature on the activity of immobilized lipase (■) and soluble lipase (●). Data points are means for three parallel measurements (SD < 10%).

Utilization of lipase enzymes in industrial processes, especially biodiesel production, often meets the challenge of thermal inactivation.  Figure 4 illustrates the similar optimal temperature for soluble lipase and lipase/PD/AgNPs complex at 40°C. We assume that the polydopamine matrix does not influence practically the activating energy of lipase hydrolysis. Significantly, at high temperatures of 45, 50, and 55°C, immobilized lipase activities were higher than those of soluble lipase from approximately 10 to 20%. It was identical to 2,4,6-trichloro-s-triazine activated polyvinyl alcohol-immobilizing porcine pancreatic lipase [46]. By means of this, our work contributes to knowledge of lipase immobilization with regards to temperature resistance since over 80% relevant works use immobilized lipases [8]. Hence, the optimal temperature 40°C was applied throughout biodiesel production experiments.

**Figure 5:**
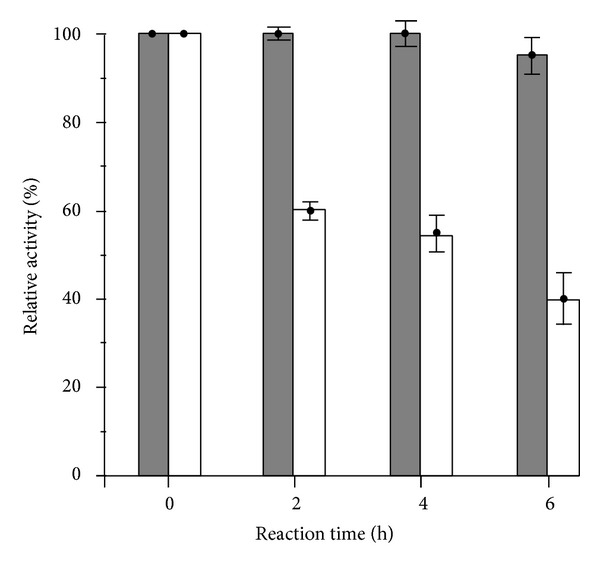
Thermal stability of immobilized lipase (dark bars) and soluble lipase (white bars) activity at 40°C. Data points are means for three parallel measurements (SD < 10%).

Thermal stability of the soluble and immobilized lipase at 40°C was consequently determined as illustrated in Figure 5. After 6-hour incubation, the immobilized lipase still maintained the high activity at 95%, whereas the native lipase activity decreased after 2 hours to 60% and dramatically dropped to 40% after 6-hour incubation. This phenomenon was similar to previous reports [18, 37, 47] in which the immobilized lipase is obviously stable in comparison to soluble native form in terms of long incubation period at high temperature. Taken into consideration, the thermal stability of LPA complex will be useful when it would have been applied to increase hydrolysis yield by extending of incubation time and to treat recalcitrant substrates, for example, waste cooking oil, in near future.

### 3.4. Conversion of Soybean Oil by LPA Complex

**Figure 6:**
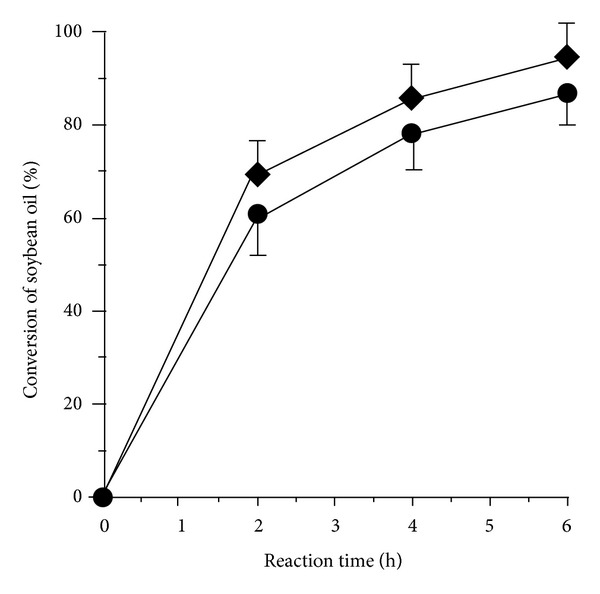
Conversion rate of soybean oil to biodiesel products at different reaction time for 6 hours by lipase/PD/AgNPs complex (◆) and soluble lipase (●). Data points are means for three parallel measurements (SD < 10%).

For the biodiesel production experiment, an approximately 10 mg of the LPA complex and 20 mg of lyophilized lipase (approximately 478 U towards the hydrolysis of Tween 80) were taken for transesterification of soybean oil with MeOH at 40°C for 6 hours (optimal conditions). The fatty acid compositions of soybean oil sample were detected including linoleic acid (50%), oleic acid (25%), palmitic acid (10%), linolenic acid (8%), and stearic acid (4%). The results showed that soybean oil conversion rate by immobilized lipase was higher than free lipase (Figure 6). GC analysis of FAMEs showed that the LPA complex could produce biodiesel product at up to 95% whereas the free lipase (lyophilized lipase was directly added to reaction mixture) could give 86% at 40°C after 6-hour reaction time. Comparatively, LPA complex was effective for biodiesel production from soybean oil when soybean oil conversion yield was 65 and 80% by using lipase from* Pseudomonas cepacia* and* Rhizomucor miehei*, respectively [48]. In other cases, the mixture of immobilized* Rhizopus oryzae* and* Candida rugosa* lipases was reported to give soybean conversion at 99% but the transesterification consumed 21 hours [49, 50]. Herein, simple covalent bonding-based immobilization technique by using polydopamine as supporting matrix is very promising to increase the biodiesel production yield since the lipase structure is probably not altered during interaction with other immobilizing compositions.

### 3.5. Reusability of Immobilized Lipase for Transesterification

**Figure 7:**
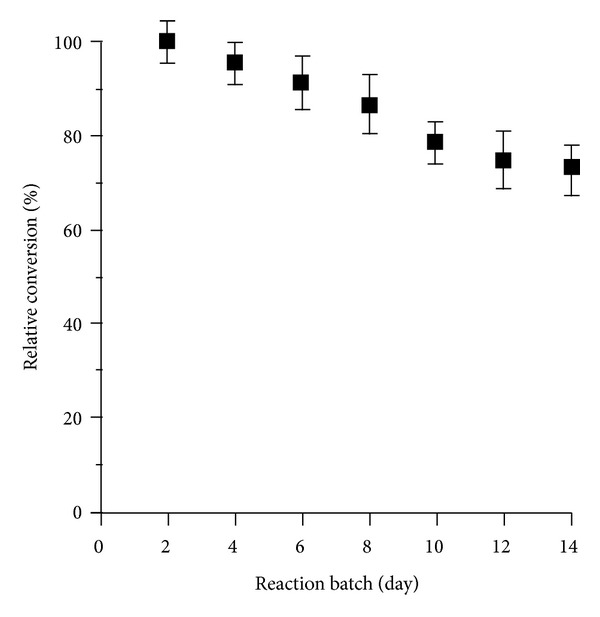
Reusability of immobilized lipase for conversion of soybean oil. Data represent maximum conversion rate of the reaction as the function of repeated batch. Data points are means for three parallel measurements (SD < 10%).

In terms of potential industrial application, the immobilization of lipase influences economic aspects not only by saving chemical reagents and reducing reaction time, but also through reusability. Remarkably, the conversion yield of soybean oil using the reused LPA complex was only decreased by approximately 27% after seven consecutive batches. The time lapse between batches was 2 days (Figure 7). The gradual decrease of oil conversion yield clearly occurred by the loss of activity of immobilized lipase. This probably happens due to the aggregation of LPA during separation by centrifugation, washing by MeOH, and lipase denaturation by heat and organic reactant (MeOH). Concerning reusability, the LPA complex was separated conveniently from the reaction batch by centrifugation and kept in sodium phosphate buffer 10 mM, pH 8.5 at 4°C without any protein preservative. Therefore, the fact of immobilization of lipase via adhesive of AgNPs and polydopamine could improve its thermal stability and show a high catalytic activity under the employed conditions. This approach could allow its utilization in transesterification of soybean oil to produce biodiesel products.

## 4. Conclusions

Biodiesel has been gaining prominence in recent years as a viable, alternative fuel to mineral-based fossil fuel diesel. The application of lipase as biocatalyst in transesterification of plant oils for biodiesel production offers an environmentally impact more attractive option compared to the conventional process. In this work, we described an alternative novel method to immobilize lipase on silver nanoparticles through an adhesive polydopamine. Our results showed that LPA complex exhibited the biodiesel products up to 95% yield. Importantly, the LPA complex displayed good reusability as well as the convenience to be centrifugally recovered.

## References

[1] Mukta N, Sreevalli Y (2010). Propagation techniques, evaluation and improvement of the biodiesel plant, Pongamia pinnata (L.) Pierre—a review. *Industrial Crops and Products*.

[2] Murphy HT, O’Connell DA, Seaton G (2012). A common view of the opportunities, challenges, and research actions for pongamia in Australia. *Bioenergy Research*.

[3] Hoffmann U (2011). Some reflections on climate change, green growth illusions and development space. *United Nations Conference on Trade and Development Discussion Papers*.

[4] Preechajarn S, Prasertsri P (2012). Biofuels Annual Bangkok Thailand 2012. *GAIN Report*.

[5] Fan X, Niehus X, Sandoval G (2012). Lipases as biocatalyst for biodiesel production. *Methods in Molecular Biology*.

[6] Kaieda M, Samukawa T, Kondo A, Fukuda H (2001). Effect of methanol and water contents on production of biodiesel fuel from plant oil catalyzed by various lipases in a solvent-free system. *Journal of Bioscience and Bioengineering*.

[7] Zhang Y, Dubé MA, McLean DD, Kates M (2003). Biodiesel production from waste cooking oil: 1. Process design and technological assessment. *Bioresource Technology*.

[8] Fjerbaek L, Christensen KV, Norddahl B (2009). A review of the current state of biodiesel production using enzymatic transesterification. *Biotechnology and Bioengineering*.

[9] Jegannathan KR, Abang S, Poncelet D, Chan ES, Ravindra P (2008). Production of biodiesel using immobilized lipase—a critical review. *Critical Reviews in Biotechnology*.

[10] Tan T, Lu J, Nie K, Deng L, Wang F (2010). Biodiesel production with immobilized lipase: a review. *Biotechnology Advances*.

[11] Salis A, Pinna M, Monduzzi M, Solinas V (2008). Comparison among immobilised lipases on macroporous polypropylene toward biodiesel synthesis. *Journal of Molecular Catalysis B: Enzymatic*.

[12] Kumari V, Shah S, Gupta MN (2007). Preparation of biodiesel by lipase-catalyzed transesterification of high free fatty acid containing oil from *Madhuca indica*. *Energy & Fuels*.

[13] Papov VV, Diamond TV, Biemann K, Waite JH (1995). Hydroxyarginine-containing polyphenolic proteins in the adhesive plaques of the marine mussel Mytilus edulis. *Journal of Biological Chemistry*.

[14] Lee H, Dellatore SM, Miller WM, Messersmith PB (2007). Mussel-inspired surface chemistry for multifunctional coatings. *Science*.

[15] Bu Y, Lee S (2012). Influence of dopamine concentration and surface coverage of Au shell on the optical properties of Au, Ag, and Ag_core_Au_shell_ nanoparticles. *ACS Applied Materials and Interfaces*.

[16] Rivera JG, Messersmith PB (2012). Polydopamine-assisted immobilization of trypsin onto monolithic structures for protein digestion. *Journal of Separation Science*.

[17] Peng H-P, Liang R-P, Zhang L, Qiu J-D (2013). Facile preparation of novel core-shell enzyme-Au-polydopamine-Fe_3_O_4_ magnetic bionanoparticles for glucose sensor. *Biosensors and Bioelectronics*.

[18] Ren Y, Rivera JG, He L, Kulkarni H, Lee D-K, Messersmith PB (2011). Facile, high efficiency immobilization of lipase enzyme on magnetic iron oxide nanoparticles via a biomimetic coating. *BMC Biotechnology*.

[19] Lee H, Rho J, Messersmith PB (2009). Facile conjugation of biomolecu les onto surfaces via mussel adhesive protein inspired coatings. *Advanced Materials*.

[20] Sileika TS, Kim H-D, Maniak P, Messersmith PB (2011). Antibacterial performance of polydopamine-modified polymer surfaces containing passive and active components. *ACS Applied Materials and Interfaces*.

[21] Veerapandian M, Yun K (2011). Functionalization of biomolecules on nanoparticles: specialized for antibacterial applications. *Applied Microbiology and Biotechnology*.

[22] Wang A-J, Liao Q-C, Feng J-J, Yan Z-Z, Chen J-R (2012). In situ synthesis of polydopamine-Ag hollow microspheres for hydrogen peroxide sensing. *Electrochimica Acta*.

[23] Sureshkumar M, Siswanto DY, Chen YC, Lee CK, Wang MJ (2013). Antibacterial and biocompatible surfaces based on dopamine autooxidized silver nanoparticles. *Journal of Polymer Science. B. Polymer Physics*.

[24] Jierwiriyapant P, Roche HF, Bottema JWT (1992). *Local Soybean Economies and Government Policies in Thailand and Indonesia*.

[25] Sangla L, Tepjun V, Pintasen S, Chanmuang A Soybean: option for alternative energy production.

[26] Solomon SD, Bahadory M, Jeyarajasingam AV, Rutkowsky SA, Boritz C (2007). Synthesis and study of silver nanoparticles. *Journal of Chemical Education*.

[27] Bradford MM (1976). A rapid and sensitive method for the quantitation of microgram quantities of protein utilizing the principle of protein dye binding. *Analytical Biochemistry*.

[28] Plou FJ, Ferrer M, Nuero OM (1998). Analysis of Tween 80 as an esterase/lipase substrate for lipolytic activity assay. *Biotechnology Techniques*.

[29] Boyer R (2000). *Modern Experimental Biochemistry*.

[30] Lee HJ, Lee SG, Oh EJ (2011). Antimicrobial polyethyleneimine-silver nanoparticles in a stable colloidal dispersion. *Colloids and Surfaces B: Biointerfaces*.

[31] Zapataa PA, Tamayob L, Páezb M, Cerdab E, Azócarb I, Rabagliatia FM (2011). Nanocomposites based on polyethylene and nanosilver particles produced by metallocenic, “in situ”, polymerization: synthesis, characterization, and antimicrobial behavior. *Europearn Polymer Journal*.

[32] Li B, Liu W, Jiang Z, Dong X, Wang B, Zhong Y (2009). Ultrathin and stable active layer of dense composite membrane enabled by poly(dopamine). *Langmuir*.

[33] Xu H, Shi X, Ma H, Lv Y, Zhang L, Mao Z (2011). The preparation and antibacterial effects of dopa-cotton/AgNPs. *Applied Surface Science*.

[34] Navarro E, Piccapietra F, Wagner B (2008). Toxicity of silver nanoparticles to *Chlamydomonas reinhardtii*. *Environmental Science & Technology*.

[35] Kim K-T, Truong L, Wehmas L, Tanguay RL (2013). Silver nanoparticle toxicity in the embryonic zebrafish is governed by particle dispersion and ionic environment. *Nanotechnology*.

[36] Dreyer DR, Miller DJ, Freeman BD, Paul DR, Bielawski CW (2012). Elucidating the structure of poly(dopamine). *Langmuir*.

[37] Wenlei X, Ning M (2009). Immobilized lipase on Fe_3_O_4_ nanoparticles as biocatalyst for biodiesel production. *Energy and Fuels*.

[38] Canilho N, Jacoby J, Pasc A (2013). Isocyanate-mediated covalent immobilization of Mucor miehei lipase onto SBA-15 for transesterification reaction. *Colloids and Surfaces B: Biointerfaces*.

[39] Dizge N, Keskinler B, Tanriseven A (2008). Covalent attachment of microbial lipase onto microporous styrene-divinylbenzene copolymer by means of polyglutaraldehyde. *Colloids and Surfaces B. Biointerfaces*.

[40] Dizge N, Keskinler B, Tanriseven A (2009). Biodiesel production from canola oil by using lipase immobilized onto hydrophobic microporous styrene-divinylbenzene copolymer. *Biochemical Engineering Journal*.

[41] Freitas L, Da Rós PCM, Santos JC, de Castro HF (2009). An integrated approach to produce biodiesel and monoglycerides by enzymatic interestification of babassu oil (*Orbinya* sp). *Process Biochemistry*.

[42] Ozmen EY, Yilma M (2009). Pretreatment of lipase with soybean oil before immobilization on beta-cyclodextrin-based polymer. *Colloids and Surfaces B: Biointerfaces*.

[43] Datta S, Christena LR, Rajaram YRS (2013). Enzyme immobilization: an overview on techniques and support materials. *3 Biotech*.

[44] Al-Zuhair S, Hasan M, Ramachandran KB (2003). Kinetics of the enzymatic hydrolysis of palm oil by lipase. *Process Biochemistry*.

[45] Wu Y, Wang Y, Luo G, Dai Y (2009). *In situ* preparation of magnetic Fe_3_O_4_-chitosan nanoparticles for lipase immobilization by cross-linking and oxidation in aqueous solution. *Bioresource Technology*.

[46] Kartal F, Kilinç A (2006). Immobilization of pancreatic lipase on polyvinyl alcohol by cyanuric chloride. *Preparative Biochemistry and Biotechnology*.

[47] Narwal SK, Saun NK, Gupta R (2014). Characterization and catalytic properties of free and silica-bound lipase: a comparative study. *Journal of Oleo Science*.

[48] Shieh C-J, Liao H-F, Lee C-C (2003). Optimization of lipase-catalyzed biodiesel by response surface methodology. *Bioresource Technology*.

[49] Lee DH, Kim JM, Shin HY, Kang SW, Kim SW (2006). Biodiesel production using a mixture of immobilized *Rhizopus oryzae* and *Candida rugosa* lipases. *Biotechnology and Bioprocess Engineering*.

[50] Lee JH, Lee DH, Lim JS (2008). Optimization of the process for biodiesel production using a mixture of immobilized *Rhizopus oryzae* and *Candida rugosa* lipases. *Journal of Microbiology and Biotechnology*.

